# One-year results of trabeculectomy with emphasis on the effect of patients’ age

**DOI:** 10.1007/s10384-024-01131-w

**Published:** 2024-10-14

**Authors:** Yuto Iwaki, Sotaro Mori, Mina Okuda-Arai, Fumio Takano, Kaori Ueda, Mari Sakamoto, Yuko Yamada-Nakanishi, Makoto Nakamura

**Affiliations:** Department of Surgery, Division of Ophthalmology, Kobe University Graduate School of Medicine, 7-5-1 Kusunoki-cho, Chuo-ku, Kobe, 650-0017 Japan

**Keywords:** Trabeculectomy, Age, Exfoliation glaucoma

## Abstract

**Purpose:**

This study investigated the association between one-year surgical outcomes following trabeculectomy and age, accounting for confounding factors.

**Study Design:**

Retrospective observational study.

**Method:**

Analyzing data from 305 patients undergoing initial trabeculectomy from 2019 onward, we employed three approaches to adjust variables: stratified analysis, regression analysis, and propensity score matching. Surgical success at 1-year post-surgery was defined by two criteria: achieving intraocular pressure of between 5 and 15 mmHg with a ≥ 20% reduction compared to pre-surgery levels and no additional glaucoma surgery (Criterion A); achieving intraocular pressure of between 5 and 12 mmHg with a ≥ 30% reduction compared to pre-surgery levels and no additional glaucoma surgery (Criterion B).

**Results:**

Stratified analysis by age unveiled a significant increase in exfoliation glaucoma (XFG) and a trend towards shorter axial lengths with advancing age (both *p* < 0.0001). Older age groups were more likely to experience surgical failure in both Criterion A and B (*p* = 0.21, < 0.01). Univariate analysis showed age as a significant factor in surgical failure for Criterion A (*p* < 0.05) and a nearly significant factor for Criterion B (*p* = 0.12). However, this trend was not evident in multivariate analysis (*p* = 0.23/0.88), where XFG became a significant factor for surgical failure (both *p* < 0.001) in Criteria A and B. Propensity score matching revealed no significant differences in surgical success rates for Criteria A and B between younger and older patients (*p* = 1.00 and 0.88).

**Conclusion:**

Age is not a primary determinant of failure in trabeculectomy; however, the increasing incidence of XFG with aging suggests a potential for poorer outcomes.

**Supplementary Information:**

The online version contains supplementary material available at 10.1007/s10384-024-01131-w.

## Introduction

As societal progress continues, the demographic landscape is shifting towards an aging population. Consequently, instances of performing trabeculectomy on elderly individuals are on the rise. A recurring challenge in this context is the observation that the conjunctiva of elderly patients tends to be thin and fragile, complicating the trabeculectomy procedure. Unlike other micro-invasive glaucoma surgery (MIGS) and tube shunt procedures, trabeculectomy requires post-operative interventions such as laser suture lysis, needling, and bleb revision surgery at the appropriate timing. Therefore, we need patient cooperation for frequent examinations. However, given the difficulty of long-term hospitalization and frequent hospital visits for older individuals, there is often hesitation in pursuing trabeculectomy. As outlined, trabeculectomy proves to be a challenging undertaking in elderly patients, yet there is no consensus on whether advanced age directly correlates with unfavorable postoperative outcomes.

Historically, young age was recognized as a poor prognostic factor for trabeculectomy. The use of mitomycin C application in trabeculectomy became commonplace before the widespread adoption of tube shunt and MIGS procedures. During that era, young age was considered a risk factor for trabeculectomy failure. A 1993 study conducted at Moorfields Eye Hospital in the UK [1] and a 2002 American study investigating trabeculectomy for 779 eyes [2] revealed that young age was a significant risk factor for surgical failure. The prevailing belief was that ideal bleb formation could not be maintained due to scar formation after trabeculectomy, given the high wound-healing capacity of young individuals.

However, recent years have witnessed a reevaluation of whether young age remains a distinct risk factor for trabeculectomy. Reports from the United States in 2006 [3] and 2012 [4] indicate that age was not a decisive risk factor for trabeculectomy. Conversely, a recent report from South Korea demonstrates an opposing trend [5]. The study unveiled a significantly lower surgical success rate in the 60 and older age group (*n* = 58) compared to the under-60 age group (*n* = 85), identifying older age as a risk factor. The Korean report acknowledges significant differences in preoperative intraocular pressure (IOP) and glaucoma disease type between the two groups, raising uncertainty about age’s true contribution to these results. Moreover, the report defines surgical success merely as IOP reduction rates, potentially skewing results in favor of the younger group with a more elevated preoperative IOP.

Studies on trabeculectomy have employed diverse definitions of surgical success. Notably, when considering age as a variable, younger patients are known to be more prone to complications related to hypotony post-trabeculectomy [6, 7]. Hence, research focusing on age should encompass hypotony as a criterion for surgical failure. Additionally, in Far-East Asian countries, including Japan, where the prevalence of normal-tension glaucoma is high [8], establishing a cutoff IOP based on a simple value is insufficient. Criteria should extend to include a condition of 20% or 30% lower than the preoperative IOP.

In light of these considerations, our study investigated the impact of age on trabeculectomy outcomes, employing surgical success criteria that account for confounding variables. Methodologies such as stratified analysis, multivariate analysis, and matching were employed to discern whether age genuinely influences trabeculectomy outcomes.

## Method

### Subjects and surgical methods

Subjects included adults aged 20 years and older who underwent initial trabeculectomy at Kobe University Hospital from January 2019 to September 2022. Exclusion criteria comprised individuals with a history of prior trabeculectomy; in cases where both eyes were treated, only the first eye to undergo surgery was included. A total of 305 eyes from 305 cases with data up to 1 year after surgery were analyzed. Surgery was conducted under sub-Tenon anesthesia using 2% lidocaine. The procedure involved creating a limbal-based conjunctival peritomy in the nasal superior quadrant, followed by the formation of a half-thickness 4 × 4-mm scleral flap. After the initial flap creation, 0.04% mitomycin C was applied for 3 min, followed by rinsing with 200 ml of balanced salt solution. Subsequently, a second scleral flap was created under the first flap, and the trabecular tissue and the second scleral were excised. A peripheral iridectomy was performed, and the first scleral flap was closed with four to seven stitches of 10 − 0 nylon sutures. Conjunctival adaptation was performed with 10 − 0 nylon sutures [9, 10].

### Evaluation parameters

Patient parameters, including age, sex, glaucoma disease type, IOP, glaucoma drug score, axial length, mean deviation of the Humphrey visual field test, and the use of anti-thrombotic medication, were investigated. The glaucoma drug score assigned 1 point for each type of eye drops, while combination drugs and oral carbonic anhydrase inhibitors were counted as 2 points [11]. Cases in which preoperative administration of anti-thrombotic medications was discontinued, were defined as not taking any anti-thrombotic drugs [12]. Information about the surgeon and whether concomitant cataract surgery was performed at the time of trabeculectomy was also collected. For dropout cases undergoing additional glaucoma surgery within one year, the value just before the reoperation was supplemented as the subsequent postoperative value using the last observation carried forward method.

### Success criteria

Two criteria were established to define surgical success: Criterion A, a loose standard, and Criterion B, a strict standard. According to Criterion A, success was defined as an IOP within 5–15 mmHg with a decrease of 20% or more from the preoperative level at 1 year after surgery, and no additional glaucoma surgery. Criterion B was defined as IOP within 5–12 mmHg with a decrease of 30% or more from preoperative levels at 1 year after surgery, and no additional glaucoma surgery. Both criteria excluded postoperative laser suture lysis, needling procedures, or bleb revision surgery as additional glaucoma surgery [12].

### Statistical analysis

**Fig. 1:**
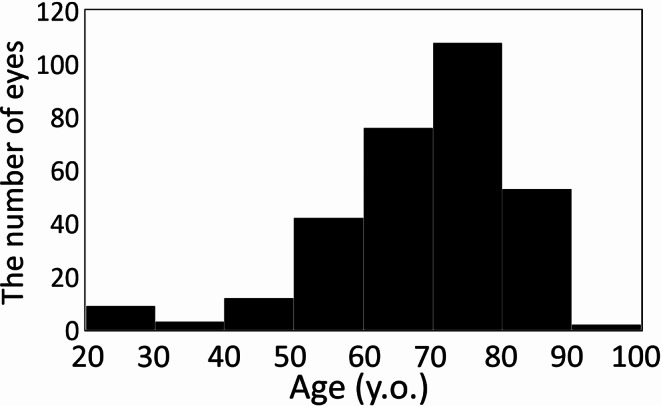
Histogram of age distribution among trabeculectomy patients

Continuous variables were expressed as the median and interquartile range in this study because the age distribution of patients who underwent trabeculectomy was non-normal (Fig. 1). Preoperative patient background changes were examined by dividing patients into four age groups (< 60, 60–69, 70–79, ≥ 80). Regression analysis was performed to determine whether age was a risk factor for surgical failure. This analysis included univariate and multivariate components, with the latter incorporating other confounding factors. Additionally, patients were divided into two groups based on median age, and differences in trabeculectomy outcomes were investigated after adjusting for confounding factors using propensity score matching. Explanatory variables for regression analysis and propensity score creation included age, glaucoma disease type, surgeon, preoperative IOP, preoperative mean deviation of the Humphrey visual field test, axial length [7], presence or absence of anti-thrombotic medication use [12], and presence or absence of concomitant cataract surgery [13]. However, age was excluded from the propensity score calculations.

### Ethics approval

This study adhered to the tenets of the Declaration of Helsinki and was approved by the Institutional Review Board of Kobe University (No. B200091). Opt-out consent was obtained from all patients. This method was used for participant recruitment in the study. Informed consent was not obtained from the patients as this study was retrospective and observational. However, patients were allowed to withdraw their consent anytime in an opt-out fashion.

## Results

Table 1 presents a comparison of the total 305 patients categorized into four age groups. In addition to age (*p* < 0.000001), the prevalence of exfoliation glaucoma significantly increased with advancing age (*p* < 0.0001). Furthermore, axial length tended to be significantly shorter in the elderly group compared to the younger group (*p* < 0.000001). Additionally, the proportion of patients taking anti-thrombotic medications increased with aging (*p* = 0.02). No significant association with age was observed in preoperative IOP, glaucoma drug score, or the degree of visual field defects. Concerning the surgical success rate, there was a tendency for surgical failure to occur with age for criteria A and B, particularly for criterion B, where the surgical success rate significantly decreased with age (*p* < 0.01).

**Table 1:** Preoperative and postoperative parameters stratified by age group

Group	< 60 (*n* = 62)	60–69 (*n* = 71)	70–79 (*n* = 108)	≥ 80 (*n* = 64)	*P* value	All (*n* = 305)
Age, yrs	52.5 (45.3, 56)	66 (63, 68)	75 (72, 77)	83 (81, 85)	**< 0.000001**	72 (62, 79)
Right Eye	35 (56.4)	34 (47.8)	46 (42.6)	26 (40.6)	0.26	141 (46.3)
Male	41 (66.1)	40 (56.3)	64 (59.3)	33 (51.5)	0.41	178 (58.4)
Glaucoma Disease Type						
Primary Open Angle Glaucoma	33 (53.2)	44 (62.0)	59 (54.6)	30 (46.9)	0.37	166 (54.4)
Exfoliation Glaucoma	3 (4.8)	9 (12.7)	26 (24.1)	24 (37.5)	**< 0.0001**	62 (20.3)
Other Secondary Glaucoma	22 (35.4)	18 (25.3)	23 (21.3)	10 (15.6)	0.59	73 (23.9)
Childhood Glaucoma	4 (6.4)	0 (0)	0 (0)	0 (0)	**< 0.01**	4 (1.3)
Preoperative IOP, mmHg	24.5 (19, 28.8)	22 (17.5, 31.5)	26.5 (20, 35)	26 (21, 34)	0.07	25 (19, 33)
Preoperative Glaucoma Drug Score	5 (4, 6)	5 (4, 5)	5 (4, 5)	4 (4, 5)	0.32	5 (4, 5)
Preoperative HVF MD value, dB	-20.25 (-12.27, -23.98)	-20.06 (-12.64, -23.85)	-18.33 (-11.75, -22.53)	-21.91 (-17.38, -26.62)	0.14	-19.51 (-12.79, -24.21)
Axial length, mm	26.25 (24.88, 27.39)	25.57 (24.24, 26.67)	24.75 (23.70, 25.59)	23.75 (23.03, 24.67)	**< 0.000001**	24.84 (23.1, 26.25)
Anti-thrombotic Medication Use	2 (3.2)	10 (14.0)	13 (12.2)	17 (26.5)	**0.02**	42 (13.7)
Concomitant Cataract Surgery	1 (1.6)	1 (1.4)	13 (12.0)	5 (7.8)	0.11	20 (6.6)
Bleb Revision	14 (22.5)	19 (26.8)	21 (19.4)	13 (20.3)	0.69	67 (22.0)
1-year IOP, mmHg	11 (8, 14)	12 (9, 16)	12 (9, 15)	12.5 (8, 17)	0.34	12 (9, 16)
1-year Glaucoma Drug Score	0 (0, 1)	0 (0, 1.5)	0 (0, 1)	0 (0, 2)	0.40	0 (0, 1)
Hypotony (< 5mmHg)	3 (4.8)	4 (5.6)	2 (1.9)	2 (3.1)	0.55	11 (3.6)
Surgical success A **(IOP ≤ 15mmHg**,** 20%)**	46 (74.2)	36 (50.7)	72 (66.7)	36 (56.3)	0.21	190 (62.2)
Surgical success B **(IOP ≤ 12mmHg**,** 30%)**	38 (61.3)	31 (43.6)	58 (53.7)	30 (46.9)	**< 0.01**	157 (51.4)

IOP: Intraocular pressure, HVF: Humphrey visual field; MD, Mean deviation. Continuous variables were shown as medians (interquartile range) and tested using the Kruskal-Wallis test. Categorical variables were shown as numbers (proportions) and tested using the chi-square test. P values in bold indicate statistically significant

Tables 2 and 3 demonstrate the results of univariate and multivariate analyses examining factors associated with cases that do not meet criteria A and B. Aging emerged as a significant factor in surgical failure for Criterion A in univariate analysis (*p* < 0.047: 95%CI 1.00-1.04), and while it exhibited a correlation with surgical failure for Criterion B, the difference was not statistically significant (*p* = 0.12: 95%CI 1.00-1.03). In univariate analysis, exfoliation glaucoma was a significant factor in surgical failure for both criteria (*p* < 0.00001 and < 0.0001). Other secondary glaucoma was a significant factor for surgical failure only in surgical success criterion B (*p* = 0.042).

**Table 2:** Factors associated with trabeculectomy failure (Criterion A)

Surgical success A	Univariate analysis		Multivariate analysis	
(IOP ≤ 15mmHg, 20%)	Odds Ratio (95%CI)	*P* value	Odds Ratio (95%CI)	*P* value
Age	1.02 (1.00-1.04)	**0.047**	1.01 (0.99–1.04)	0.23
Axial Length	0.96 (0.85–1.08)	0.51	1.02 (0.88–1.17)	0.51
Preoperative IOP	1.01 (0.98–1.03)	0.57	0.99 (0.96–1.02)	0.44
Preoperative HVF MD value	1.01 (0.98–1.04)	0.65	1.00 (0.97–1.04)	0.90
Concomitant Cataract Surgery	1.11 (0.44–2.80)	0.83	1.00 (0.37–2.72)	0.99
Anti-thrombotic Medicine Use	1.02 (0.52–1.99)	0.96	0.80 (0.38, 1.70)	0.57
Surgeon				
Surgeon B to A	0.91 (0.40–2.05)	0.82	0.87 (0.36–2.11)	0.76
Surgeon C to A	1.02 (0.55–1.89)	0.96	0.95 (0.48–1.86)	0.88
Surgeon D to A	1.41 (0.75–2.65)	0.29	1.28 (0.66–2.51)	0.57
Glaucoma Disease Type				
Exfoliation Glaucoma to POAG	4.42 (2.39–8.20)	**< 0.00001**	4.41 (2.27–8.54)	**< 0.0001**
Other Secondary Glaucoma to POAG	1.72 (0.97–3.07)	0.067	1.85 (0.98–3.49)	0.059
Childhood Glaucoma to POAG	0.87 (0.11–8.57)	0.91	1.72 (0.14–21.90)	0.67

IOP: Intraocular pressure, CI, Confidence interval, HVF: Humphrey visual field; MD, Mean deviation; POAG, Primary open angle glaucoma. P values in bold indicate statistically significant

**Table 3:** Factors associated with trabeculectomy failure (Criterion B)

Surgical success B	Univariate analysis		Multivariate analysis	
(IOP ≤ 12mmHg, 30%)	Odds Ratio (95%CI)	*P* value	Odds Ratio (95%CI)	*P* value
Age	1.01 (1.00-1.03)	0.12	1.00 (0.98–1.02)	0.88
Axial Length	0.93 (0.82–1.04)	0.20	0.97 (0.85–1.11)	0.66
Preoperative IOP	1.00 (0.98–1.03)	0.77	0.99 (0.96–1.02)	0.30
Preoperative HVF MD value	1.01 (0.98–1.04)	0.60	1.01 (0.98–1.04)	0.64
Concomitant Cataract Surgery	2.06 (0.80–5.32)	0.13	1.69 (0.62–4.62)	0.31
Anti-thrombotic Medicine Use	1.49 (0.78–2.88)	0.23	1.24 (0.61–2.55)	0.55
Surgeon				
Surgeon B to A	0.91 (0.41–1.98)	0.81	0.90 (0.39–2.08)	0.80
Surgeon C to A	1.38 (0.76–2.51)	0.29	1.31 (0.69–2.48)	0.42
Surgeon D to A	1.46 (0.79–2.71)	0.23	1.36 (0.71–2.61)	0.35
Glaucoma Disease Type				
Exfoliation Glaucoma to POAG	3.52 (1.89–6.56)	**< 0.0001**	3.56 (1.83–6.93)	**< 0.001**
Other Secondary Glaucoma to POAG	1.78 (1.02–3.11)	**0.042**	1.88 (1.02–3.48)	**0.043**
Childhood Glaucoma to POAG	0.52 (0.05–5.09)	0.57	0.73 (0.06–8.83)	0.80

IOP: Intraocular pressure, HVF: Humphrey visual field; MD, Mean deviation; POAG, Primary open angle glaucoma. P values in bold indicate statistically significant

In multivariate analysis, age ceased to be a significant factor for trabeculectomy failure (*p* = 0.23 and 0.88). Conversely, exfoliation glaucoma remained a significant risk factor after multivariate analysis (*p* < 0.0001 and < 0.001). Other secondary glaucoma was a significant factor only in Criterion B in multivariate (*p* = 0.043) as well as univariate analysis. Furthermore, in both univariate and multivariate analyses, axial length did not emerge as a significant factor related to surgical success (Criterion A: both *p* = 0.51, Criterion B: *p* = 0.20 and 0.66).

Table 4 shows the surgical success rate results of the total 305 cases divided into two groups—the younger and the elderly group—based on the median age of 72 years. Preoperative background factors, including age, showed significant differences between the two groups, along with glaucoma disease type, IOP, axial length, the proportion of anti-thrombotic medication use, and the rate of concomitant cataract surgery. Therefore, using these raw data to accurately determine the effect of age proved challenging.

**Table 4:** Comparison between young and elderly groups using all data

	< 72 (*n* = 153)	≥ 72 (*n* = 152)	*P* value
Age, yrs	62 (55, 68)	79 (75, 82.3)	**< 0.000001**
Right Eye	75 (49.0)	66 (43.4)	0.36
Male	97 (63.4)	81 (53.3)	0.08
Glaucoma Disease Type			
Primary Open Angle Glaucoma	92 (60.1)	74 (48.7)	0.05
Exfoliation Glaucoma	14 (9.2)	48 (31.6)	**< 0.000001**
Other Secondary Glaucoma	43 (28.1)	30 (19.7)	0.11
Childhood Glaucoma	4 (2.6)	0 (0)	0.12
Preoperative IOP mmHg	23 (18, 32)	26 (21, 34)	**0.01**
Preoperative Glaucoma Drug Score	5 (4, 5)	5 (4, 5)	0.18
Preoperative HVF MD value, dB	-19.51 (-12.09, -24.08)	-19.52 (-13.89, -25.01)	0.54
Axial length, mm	25.73 (24.44, 27.06)	24.32 (23.36, 25.28)	**< 0.00001**
Anti-thrombotic Medication Use	13 (8.4)	29 (19.1)	**< 0.01**
Concomitant Cataract Surgery	3 (2.0)	17 (11.2)	**< 0.01**
Bleb Revision	35 (22.9)	32 (21.1)	0.41
1-year IOP, mmHg	11 (8, 15)	12 (9, 16)	0.07
1-year Glaucoma Drug Score	0 (0, 1)	0 (0, 2)	0.17
Hypotony (< 5mmHg)	7 (4.6)	4 (2.6)	0.54
Surgical success A (IOP ≤ 15mmHg, 20%)	98 (64.1)	92 (60.5)	0.56
Surgical success B (IOP ≤ 12mmHg, 30%)	82 (53.6)	75 (49.3)	0.49

IOP: Intraocular pressure, HVF: Humphrey visual field; MD, Mean deviation. The Mann-Whitney test was used for continuous variables, while the Fisher exact test was used for binary variables. P values in bold indicate statistically significant

Table 5 presents the analysis results after adjusting for confounding factors using the propensity score matching method. Differences in preoperative background factors, except for age, were mitigated between the two groups. Additionally, the surgical success rate showed no statistically significant differences between the two groups under both criteria, even after adjusting for the confounding factors.

**Table 5:** Comparison between young and elderly groups after propensity score matching

	< 72 (*n* = 82)	≥ 72 (*n* = 82)	*P* value
Age, yrs	62 (56, 69)	78 (75, 81.8)	**< 0.00001**
Right Eye	37 (45.1)	36 (43.9)	1.00
Male	52 (63.4)	45 (54.9)	0.27
Glaucoma Disease Type			
Primary Open Angle Glaucoma	47 (57.3)	44 (53.7)	0.64
Exfoliation Glaucoma	13 (15.9)	16 (19.5)	0.68
Other Secondary Glaucoma	22 (26.8)	22 (26.8)	1.00
Childhood Glaucoma	0 (0)	0 (0)	1.00
Preoperative IOP, mmHg	24 (17.3, 34.5)	25.5 (20, 33.8)	0.46
Preoperative Glaucoma Drug Score	5 (4, 5.8)	5 (4, 5)	0.32
Preoperative HVF MD value, dB	-20.69 (-11.5, -25.09)	-19.46 (-11.73, -23.62)	0.78
Axial length, mm	24.75 (23.67, 25.78)	24.77 (23.87, 25.94)	0.77
Anti-thrombotic Medication Use	10 (12.2)	12 (14.6)	0.82
Concomitant Cataract Surgery	3 (3.7)	5 (6.1)	0.72
Bleb Revision	19 (23.2)	18 (22.0)	0.85
1-year IOP, mmHg	12 (9, 15)	12 (8.3, 16)	0.92
1-year Glaucoma Drug Score	0 (0, 2)	0 (0, 2)	0.93
Hypotony (< 5mmHg)	1 (1.2)	1 (1.2)	1.00
Surgical success A (IOP ≤ 15mmHg, 20%)	54 (65.9)	55 (67.1)	1.00
Surgical success B (IOP ≤ 12mmHg, 30%)	43 (52.4)	45 (54.9)	0.88

IOP: Intraocular pressure, HVF: Humphrey visual field; MD, Mean deviation. The Mann-Whitney test was used for continuous variables, while the Fisher exact test was used for binary variables. P values in bold indicate statistically significant

## Discussion

In our examination of the relationship between age and trabeculectomy success through simple age-based comparisons and univariate regression, a notable trend emerged—older patients were more likely to experience trabeculectomy failure. These findings align with recent reports from South Korea, a region with a genetic background similar to that of Japan [5].

However, when we conducted analyses utilizing multiple regression analysis and propensity score matching to account for confounding factors, age ceased to be a primary risk factor for trabeculectomy failure. Instead, we observed that the prevalence of exfoliation glaucoma increased with aging, leading to poorer outcomes in the elderly. A previous report from South Korea [5], echoing our findings, categorizes exfoliation glaucoma as “Other secondary glaucoma” and highlights the risks associated with advanced age. However, the extent to which exfoliation glaucoma played a role in that report remains unclear. The authors did note differences in bleb height and vascularity when comparing elderly and young individuals, suggesting that elderly patients tend to have flatter bleb height and increased vascularity. We speculate that this characteristic may be related to exfoliation glaucoma. Indeed, a previous report from Sweden reveals that trabeculectomy outcomes for exfoliation glaucoma are significantly worse than for primary open-angle glaucoma (POAG), emphasizing smaller bleb height and extent along with increased vascularity in exfoliation glaucoma [14].

Exfoliation glaucoma, characterized by increased IOP due to extracellular deposits, becomes more prevalent with age [15]. Previous research proposes that the poor surgical outcomes in trabeculectomy for exfoliation glaucoma may be attributed to the extracellular material obstructing the pathway created during surgery. This pathway flows from the anterior chamber to the subconjunctiva through the peripheral iridectomy [14]. Additionally, studies report higher flare values in the anterior chamber after trabeculectomy in exfoliation glaucoma patients compared to POAG patients, and this increased postoperative inflammation has been identified as a potential cause of trabeculectomy failure [16]. Ayala et al. propose that the suboptimal performance of trabeculectomy in exfoliation glaucoma may be attributed to the advanced age of patients due to a lengthier exposure to glaucoma eye drops [14]. The prolonged use of these eye drops is posited to elevate the risk of inflammation, potentially exacerbated by additives like benzalkonium chloride present in the eye drops [17]. This observation aligns with our earlier findings, wherein an extended history of glaucoma eye drop utilization was associated with suboptimal postoperative outcomes in MIGS [18]. However, contrary to our previous study, the current investigation indicates that age no longer holds prominence as the primary risk factor for surgical outcomes in trabeculectomy. Thus, it is suggested that an extended history of eye drop use may not be a definitive determinant of poor outcomes in trabeculectomy, as demonstrated in this study.

Why do earlier reports, from before the early 2000s contend that the results of trabeculectomy are worse in younger patients? It is evident that the influence of hypotony, particularly its higher frequency in young individuals, played a significant role. However, this cannot explain why, in recent years young age is no longer considered a risk factor in trabeculectomy. We posit that this persistence may be attributed to the relatively high incidence of neovascular glaucoma (NVG) in young patients. In the era when tube shunt surgery was not prevalent, trabeculectomy stood as the primary option for NVG. However, with the increased effectiveness of tube shunts for NVG [19], tube shunt surgery is now often chosen as the primary choice. Consequently, recent reports are unlikely to report trabeculectomy failure in young individuals.

Our results reveal a surprising trend in which axial length tended to decrease in the elderly group. Myopia is a well-known risk factor for glaucoma patients [20]. We hypothesize that glaucoma patients affected by myopia might experience worsened glaucoma at a young age, leading to longer axial length in patients undergoing trabeculectomy at a younger age. Conversely, a survey of Japanese glaucoma patients indicates that the emmetropic or hyperopic group was more likely to experience worsening visual field defects than the myopic group [21]. Given that glaucoma deterioration is more common in the elderly, axial length tends to be shorter in the elderly group in the current study. However, this axial length was not associated with the trabeculectomy success rate. Previous reports indicate that eyes with a long axial length are more prone to developing hypotony maculopathy after trabeculectomy [22]. In the current study, with only 11 cases (3.6%) of hypotony among 305 eyes, the results may not have reached significance.

This study employed various analysis methods—multiple regression analysis and propensity score—in addition to simpler methods such as stratified analysis and univariate regression analysis to explore the impact of age on trabeculectomy outcomes. Simple analyses run the risk of overlooking confounding factors that may exist in the background. Association does not imply causation, as seen in the example of blood pressure and hair volume outcomes being correlated due to a hidden common cause, age. It is crucial to consider underlying confounds.

Two primary approaches exist to adjust for confounding factors: one measures the influence of confounding factors, and the other aligns all confounding factors. The former is multivariate regression analysis, while the latter is the propensity score method [23]. Multivariate regression analysis is useful when the exposure variable is continuous or a multigroup categorical variable. However, it has the disadvantage of less intuitive results’ interpretation and potential overfitting when numerous confounding factors need consideration [24]. On the other hand, the propensity score method allows consideration of an unlimited number of confounds and provides a clear understanding, as demonstrated in Table 5, but it is less popular than multivariate regression analysis and involves more analysis steps, making calculations cumbersome and time-consuming. The practice of verifying results’ robustness by using different statistical models is termed sensitivity analysis [25]. Through this comprehensive statistical approach, we determined that age is not a primary factor influencing surgery, but the rate of exfoliation glaucoma increases with aging, leading to poor outcomes in the elderly group.

To further strengthen the results of this study, a subanalysis was conducted excluding all cases of exfoliation glaucoma. As shown in Supplemental Table 1, the trend of increasing surgical failure with age disappeared in this dataset. In this study, bleb revision was not defined as a surgical failure. However, because some consider bleb revision to be a reoperation, we reanalyzed the data, considering bleb revision as a surgical failure. These results are shown in Supplemental Tables 2, 3, and 4. In this reanalysis, age was not a factor in the failure of trabeculectomy, but exfoliation glaucoma was.

There are several limitations to this study. First, the study was conducted at a single institution and included a biased population of only Japanese patients. In addition, the results are for short-term outcomes one year after surgery, and future studies will be required to determine long-term outcomes. Although the analysis focused on age, it is important to note that this dataset does not include a large number of patients under the age of 40.

In conclusion, age itself does not emerge as a primary factor indicating poor surgical outcomes for trabeculectomy. Instead, the prevalence of exfoliation glaucoma increases with age, resulting in inferior trabeculectomy results.

## Electronic supplementary material

Below is the link to the electronic supplementary material.

Supplementary Material 1

Supplementary Material 2

Supplementary Material 3

Supplementary Material 4
