# Supplementary material for: One-year results of trabeculectomy with emphasis on the effect of patients’ age
Source: Jpn J Ophthalmol. 2024 Oct 14;69(1):93–100. doi: 10.1007/s10384-024-01131-w (PMC11821737; doi:10.1007/s10384-024-01131-w)
Supplement: Supplementary file 1 — Supplementary Material 1 [file 10384_2024_1131_MOESM1_ESM.docx]

**Supplemental Table 1.** Preoperative and postoperative parameters stratified by age group without exfoliation glaucoma dataset.

| **Group** | **<60** (n=59) | **60-69** (n=62) | **70-79** (n=82) | **≥80** (n=40) | ***P* value** | **All** (n=243) |
| --- | --- | --- | --- | --- | --- | --- |
| **Age, yrs** | 52 (45, 56) | 66 (63, 68) | 74 (72,77) | 83 (81,85) | **<0.000001** | 70 (60,77) |
| **Right Eye** | 34 (57.6) | 28 (45.2) | 35 (42.7) | 15 (37.5) | 0.19 | 112 (46.1) |
| **Male** | 38 (64.4) | 35 (56.5) | 49 (60.0) | 22 (55.0) | 0.76 | 144 (59.3) |
| **Glaucoma Disease Type** |  |  |  |  |  |  |
| **Primary Open Angle Glaucoma** | 33 (56.0) | 44 (71.0) | 59 (72.0) | 30 (75.0) | 0.13 | 166 (68.3) |
| **Other Secondary Glaucoma** | 22 (37.3) | 18 (29.0) | 23 (28.0) | 10 (25.0) | 0.54 | 73 (30.0) |
| **Childhood Glaucoma** | 4 (6.8) | 0 (0) | 0 (0) | 0 (0) | **<0.01** | 4 (1.6) |
| **Preoperative IOP, mmHg** | 24 (19, 28.5) | 22 (18, 29.5) | 25 (20,32.8) | 24.5 (20.8,32.3) | 0.34 | 24 (19, 31.5) |
| **Preoperative Glaucoma Drug Score** | 5 (4, 6) | 5 (4, 5) | 5 (4, 5) | 5 (4, 5) | 0.53 | 5 (4, 5) |
| **Preoperative HVF MD value, dB** | -20.38 (-12.44, -24.43) | -18.14 (-11.68, -23.94) | -19.25 (-13.03,-22.59) | -21.91 (-16.63,-26.71) | 0.22 | -20.05 (-12.77, -24.49) |
| **Axial length, mm** | 26.28 (25.24, 27.38) | 25.63 (24.32, 26.67) | 24.74 (23.68,25.61) | 23.79 (22.92,24.46) | **<0.000001** | 25.03 (23.82, 26.34) |
| **Anti-thrombotic Medication Use** | 2 (3.4) | 9 (14.5) | 8 (10.0) | 8 (20.0) | **0.05** | 27 (11.1) |
| **Concomitant Cataract Surgery** | 0 (0) | 1 (1.6) | 10 (12.2) | 4 (10.0) | **<0.01** | 15 (6.2) |
| **Bleb Revision** | 14 (23.7) | 12 (19.4) | 19 (23.2) | 11 (27.5) | 0.82 | 56 (23.0) |
| **1-year IOP, mmHg** | 11 (8, 14) | 12 (7.3, 15) | 12 (9,14) | 10.5 (8,14) | 0.61 | 11 (8, 14) |
| **1-year Glaucoma Drug Score** | 0 (0, 1) | 0 (0, 1) | 0 (0, 1) | 0 (0, 1) | 0.91 | 0 (0, 1) |
| **Hypotony (<5mmHg)** | 3 (5.0) | 4 (6.5) | 1 (1.2) | 0 (0) | 0.17 | 8 (3.3) |
| **Surgical success A**  **(IOP≤15mmHg, 20%)** | 45 (76.3) | 34 (54.8) | 59 (72.0) | 29 (72.5) | 0.051 | 167 (68.7) |
| **Surgical success B**  **(IOP≤12mmHg, 30%)** | 37 (62.7) | 29 (46.8) | 46 (56.1) | 26 (65.0) | 0.21 | 138 (56.8) |

IOP: Intraocular pressure, HVF: Humphrey visual field; MD, Mean deviation. Continuous variables were shown as medians (interquartile range) and tested using the Kruskal-Wallis test. Categorical variables were shown as numbers (proportions) and tested using the chi-square test. P values in bold indicate statistically significant.
