# Supplementary material for: One-year results of trabeculectomy with emphasis on the effect of patients’ age
Source: Jpn J Ophthalmol. 2024 Oct 14;69(1):93–100. doi: 10.1007/s10384-024-01131-w (PMC11821737; doi:10.1007/s10384-024-01131-w)
Supplement: Supplementary file 2 — Supplementary Material 2 [file 10384_2024_1131_MOESM2_ESM.docx]

**Supplemental Table 2.** Preoperative and postoperative parameters stratified by age group When bleb revision is considered a surgical failure.

| **Group** | **<60** (n=62) | **60-69** (n=71) | **70-79** (n=108) | **≥80** (n=64) | ***P* value** | **All** (n=305) |
| --- | --- | --- | --- | --- | --- | --- |
| **Age, yrs** | 52.5 (45.3, 56) | 66 (63, 68) | 75 (72, 77) | 83 (81, 85) | **<0.000001** | 72 (62, 79) |
| **Right Eye** | 35 (56.4) | 34 (47.8) | 46 (42.6) | 26 (40.6) | 0.26 | 141 (46.3) |
| **Male** | 41 (66.1) | 40 (56.3) | 64 (59.3) | 33 (51.5) | 0.41 | 178 (58.4) |
| **Glaucoma Disease Type** |  |  |  |  |  |  |
| **Primary Open Angle Glaucoma** | 33 (53.2) | 44 (62.0) | 59 (54.6) | 30 (46.9) | 0.37 | 166 (54.4) |
| **Exfoliation Glaucoma** | 3 (4.8) | 9 (12.7) | 26 (24.1) | 24 (37.5) | **<0.0001** | 62 (20.3) |
| **Other Secondary Glaucoma** | 22 (35.4) | 18 (25.3) | 23 (21.3) | 10 (15.6) | 0.59 | 73 (23.9) |
| **Childhood Glaucoma** | 4 (6.4) | 0 (0) | 0 (0) | 0 (0) | **<0.01** | 4 (1.3) |
| **Preoperative IOP, mmHg** | 24.5 (19, 28.8) | 22 (17.5, 31.5) | 26.5 (20, 35) | 26 (21, 34) | 0.07 | 25 (19, 33) |
| **Preoperative Glaucoma Drug Score** | 5 (4, 6) | 5 (4, 5) | 5 (4, 5) | 4 (4, 5) | 0.32 | 5 (4, 5) |
| **Preoperative HVF MD value, dB** | -20.25 (-12.27, -23.98) | -20.06 (-12.64, -23.85) | -18.33 (-11.75, -22.53) | -21.91 (-17.38, -26.62) | 0.14 | -19.51 (-12.79, -24.21) |
| **Axial length, mm** | 26.25 (24.88, 27.39) | 25.57 (24.24, 26.67) | 24.75 (23.70, 25.59) | 23.75 (23.03, 24.67) | **<0.000001** | 24.84 (23.1, 26.25) |
| **Anti-thrombotic Medication Use** | 2 (3.2) | 10 (14.0) | 13 (12.2) | 17 (26.5) | **0.02** | 42 (13.7) |
| **Concomitant Cataract Surgery** | 1 (1.6) | 1 (1.4) | 13 (12.0) | 5 (7.8) | 0.11 | 20 (6.6) |
| **Bleb Revision** | 14 (22.5) | 19 (26.8) | 21 (19.4) | 13 (20.3) | 0.69 | 67 (22.0) |
| **1-year IOP, mmHg** | 11 (8, 14) | 12 (9, 16) | 12 (9, 15) | 12.5 (8, 17) | 0.34 | 12 (9, 16) |
| **1-year Glaucoma Drug Score** | 0 (0, 1) | 0 (0, 1.5) | 0 (0, 1) | 0 (0, 2) | 0.40 | 0 (0, 1) |
| **Hypotony (<5mmHg)** | 3 (4.8) | 4 (5.6) | 2 (1.9) | 2 (3.1) | 0.55 | 11 (3.6) |
| **Surgical success A’**  **(IOP≤15mmHg, 20%)** | 39 (63.0) | 27 (38.0) | 56 (51.9) | 33 (51.6) | **0.04** | 155 (50.8) |
| **Surgical success B’**  **(IOP≤12mmHg, 30%)** | 32 (51.6) | 23 (32.4) | 44 (40.7) | 28 (43.8) | 0.16 | 127 (41.6) |

IOP: Intraocular pressure, HVF: Humphrey visual field; MD, Mean deviation. Continuous variables were shown as medians (interquartile range) and tested using the Kruskal-Wallis test. Categorical variables were shown as numbers (proportions) and tested using the chi-square test. P values in bold indicate statistically significant.
