# Supplementary material for: One-year results of trabeculectomy with emphasis on the effect of patients’ age
Source: Jpn J Ophthalmol. 2024 Oct 14;69(1):93–100. doi: 10.1007/s10384-024-01131-w (PMC11821737; doi:10.1007/s10384-024-01131-w)
Supplement: Supplementary file 3 — Supplementary Material 3 [file 10384_2024_1131_MOESM3_ESM.docx]

**Supplemental Table 3.** Factors associated with trabeculectomy failure (Criterion A’) When bleb revision is considered a surgical failure

| **Surgical success A’** | **Univariate Analysis** | | **Multivariate Analysis** | |
| --- | --- | --- | --- | --- |
| **(IOP≤15mmHg, 20%)** | **Odds Ratio (95%CI)** | ***P* value** | **Odds Ratio**  **(95%CI)** | ***P* value** |
| **Age** | 1.01 (1.00-1.03) | 0.12 | 1.01 (0.99-1.03) | 0.52 |
| **Axial Length** | 0.98 (0.88-1.11) | 0.79 | 1.02 (0.89-1.17) | 0.74 |
| **Preoperative IOP** | 0.99 (0.97-1.02) | 0.58 | 0.98 (0.96-1.01) | 0.17 |
| **Preoperative HVF MD value** | 1.00 (0.97-1.03) | 0.84 | 1.00 (0.97-1.03) | 0.88 |
| **Concomitant Cataract Surgery** | 0.84 (0.34-2.08) | 0.699 | 0.75 (0.28-2.00) | 0.57 |
| **Anti-thrombotic Medicine Use** | 0.83 (0.43-1.60) | 0.58 | 0.69 (0.34, 1.42) | 0.32 |
| **Surgeon** |  |  |  |  |
| **Surgeon B to A** | 0.91 (0.42-1.97) | 0.8 | 0.89 (0.39-2.05) | 0.79 |
| **Surgeon C to A** | 1.29 (0.71-2.34) | 0.4 | 1.29 (0.69-2.44) | 0.43 |
| **Surgeon D to A** | 1.20 (0.65-2.22) | 0.57 | 1.13 (0.69-2.14) | 0.72 |
| **Glaucoma Disease Type** |  |  |  |  |
| **Exfoliation Glaucoma to POAG** | 2.88 (1.55-5.36) | **<0.001** | 3.31 (1.69-6.48) | **<0.001** |
| **Other Secondary Glaucoma to POAG** | 1.05 (0.60-1.83) | 0.86 | 1.21 (0.66-2.23) | 0.53 |
| **Childhood Glaucoma to POAG** | 0.43 (0.04-4.17) | 0.46 | 0.68 (0.06-8.21) | 0.76 |

IOP: Intraocular pressure, CI, Confidence interval, HVF: Humphrey visual field; MD, Mean deviation; POAG, Primary open angle glaucoma. P values in bold indicate statistically significant.
