# Supplementary material for: One-year results of trabeculectomy with emphasis on the effect of patients’ age
Source: Jpn J Ophthalmol. 2024 Oct 14;69(1):93–100. doi: 10.1007/s10384-024-01131-w (PMC11821737; doi:10.1007/s10384-024-01131-w)
Supplement: Supplementary file 4 — Supplementary Material 4 [file 10384_2024_1131_MOESM4_ESM.docx]

**Supplemental Table 4.** Factors associated with trabeculectomy failure (Criterion B’) when bleb revision is considered a surgical failure

| **Surgical success B’** | **Univariate Analysis** | | **Multivariate Analysis** | |
| --- | --- | --- | --- | --- |
| **(IOP≤12mmHg, 30%)** | **Odds Ratio**  **(95%CI)** | ***P* value** | **Odds Ratio**  **(95%CI)** | ***P* value** |
| **Age** | 1.01 (0.99-1.03) | 0.27 | 1.00 (0.97-1.02) | 0.64 |
| **Axial Length** | 0.93 (0.83-1.05) | 0.24 | 0.96 (0.84-1.10) | 0.59 |
| **Preoperative IOP** | 0.99 (0.97-1.01) | 0.36 | 0.98 (0.95-1.00) | 0.08 |
| **Preoperative HVF MD value** | 1.00 (0.97-1.03) | 0.98 | 1.01 (0.98-1.04) | 0.69 |
| **Concomitant Cataract Surgery** | 1.72 (0.64-4.61) | 0.28 | 1.39 (0.49-3.92) | 0.54 |
| **Anti-thrombotic Medicine Use** | 1.34 (0.68-2.63) | 0.40 | 1.13 (0.54-2.34) | 0.75 |
| **Surgeon** |  |  |  |  |
| **Surgeon B to A** | 0.95 (0.44-2.05) | 0.89 | 0.97 (0.43-2.22) | 0.95 |
| **Surgeon C to A** | 1.75 (0.95-3.21) | 0.07 | 1.74 (0.91-3.32) | 0.09 |
| **Surgeon D to A** | 1.22 (0.66-2.26) | 0.54 | 1.17 (0.61-2.22) | 0.64 |
| **Glaucoma Disease Type** |  |  |  |  |
| **Exfoliation Glaucoma to POAG** | 2.49 (1.30-4.74) | **<0.01** | 2.89 (1.44-5.82) | **<0.01** |
| **Other Secondary Glaucoma to POAG** | 1.17 (0.67-2.04) | 0.58 | 1.32 (0.71-2.44) | 0.38 |
| **Childhood Glaucoma to POAG** | 0.29 (0.03-2.83) | 0.29 | 0.32 (0.026-3.93) | 0.38 |

IOP: Intraocular pressure, HVF: Humphrey visual field; MD, Mean deviation; POAG, Primary open angle glaucoma. P values in bold indicate statistically significant.
